# Optimal cerebral perfusion pressure during induced hypertension and its impact on delayed cerebral infarction and functional outcome after subarachnoid hemorrhage

**DOI:** 10.1038/s41598-024-82507-3

**Published:** 2024-12-16

**Authors:** Beate Kranawetter, Sheri Tuzi, Onnen Moerer, Dorothee Mielke, Veit Rohde, Vesna Malinova

**Affiliations:** 1 Department of Neurosurgery, University Medical Center Göttingen, Robert-Koch Straße 40, 37075 Göttingen, Germany; 2 Department of Anesthesiology and Intensive Care, University Medical Center Göttingen, Göttingen, Germany; 3 Department of Neurosurgery, University Hospital Ausgburg, Augsburg, Germany

**Keywords:** Optimal cerebral perfusion pressure, Subarachnoid hemorrhage, Perfusion pressure index, Induced hypertension, Cerebrovascular disorders, Medical research

## Abstract

Disturbed cerebral autoregulation (CA) increases the dependency of cerebral blood flow (CBF) on cerebral perfusion pressure (CPP). Thus, induced hypertension (IHT) is used to prevent secondary ischemic events. The pressure reactivity index (PRx) assesses CA and can determine the optimal CPP (CPPopt). This study investigates CPPopt in patients with subarachnoid hemorrhage (SAH) treated with IHT and its impact on delayed cerebral infarction and functional outcome. This is a retrospective observational study including SAH patients treated between 2012 and 2020. PRx defines the correlation coefficient of intracranial pressure (ICP) and the mean arterial pressure (MAP). The CPP corresponding to the lowest PRx-value describes CPPopt. Primary outcome parameters were deleayed cerebral infarction and functional outcome. In patients without IHT, higher deviations of measured CPP from CPPopt were associated with delayed cerebral infarction (*p* = 0.001). Longer time spent with a CPP below the calculated CPPopt during IHT led to an increased risk of developing delayed cerebral infarction (*r* = 0.39, *p* = 0.002). A larger deviation of measured CPP from CPPopt correlated with an unfavorable outcome in patients treated with IHT (*p* = 0.04) and without IHT (*p* = 0.0007). Patients with severe aneurysmal SAH may benefit from an individualized CPP management and the calculation of CPPopt may help to guide IHT.

## Introduction

Four mechanisms regulating cerebral blood flow (CBF) are commonly described in the literature: (1) neurogenic responses driven by astrocytes and microglia releasing neurotransmitters with vasoactive properties^1^; (2) metabolic mechanisms mainly regulated by pH, paCO2, and paO2 ^2^; (3) endothelial mechanisms, which rely on vasodilators such as nitric oxide (NO) and vasoconstrictors such as thromboxane A2 and endothelin-1 ^3^; and (4) myogenic tone or cerebral microvascular reactivity, which describes the ability of arterioles and small artery smooth muscle cells to contract or dilate in response to increased or decreased cerebral perfusion pressure (CPP)^4^. The last mechanism, also termed cerebral autoregulation (CA), enables the cerebral circulation to maintain a relatively stable CBF despite large changes in blood pressure^5–8^. Several cerebral pathologies, including traumatic brain injury (TBI), ischemic stroke, and aneurysmal subarachnoid hemorrhage (aSAH), can alter CA^9^. Disturbances in CA after SAH have been associated with an increased risk of delayed cerebral infarction and poor outcomes^8,10,11^. Impaired CA leads to an increased dependency of CBF on CPP, whereas insufficient CPP can lead to cerebral ischemia. Current guidelines do not recommend a specific CPP cutoff to prevent hypoperfusion for patients with aSAH to prevent delayed cerebral infarction^12^. Hemodynamic augmentation i.e. induced hypertension (IHT) by the administration of vasopressors with a targeted systolic arterial blood pressure of 180 mmHg, is widely used to improve cerebral perfusion in patients with aSAH and delayed cerebral infarction^12^. However, IHT failed to improve the outcome and is often accompanied by adverse events, bearing the risk of worsening prognosis^13^. Furthermore, the degree of CA impairment shows a significant level of variability among individuals, which questions the concept of setting a general CPP threshold for patients with aSAH and highlights the need to target the CPP at which the CA functions at its best, referred to as optimal CPP (CPPopt)^14^. The dynamic relationship between intracranial pressure (ICP) and mean arterial pressure (MAP) provides information about cerebrovascular pressure reactivity, referred to as the pressure reactivity index (PRx), which allows for the evaluation of CA^15^. PRx was calculated to determine CPPopt, which is equivalent to the CPP at the lowest level of PRx^15^. Although PRx has often been used to guide CPPopt-targeted therapy in patients with traumatic brain injury^16–18^, studies evaluating this concept in the setting of aSAH are underrepresented^19–21^. Furthermore, the interaction between CPPopt and IHT has not been clarified. This study aimed to investigate the effects of a CPPopt-guided therapy during IHT on the incidence of delayed cerebral infarction and functional outcomes after aSAH.

## Materials and methods

### Patent population

This retrospective observational study included a consecutive cohort of patients with aSAH treated at our institution between 2012 and 2020. The inclusion criteria were age ≥ 18 years, confirmed aSAH, invasive ICP monitoring, and invasive ABP measurement. Patients younger than 18 years, those without invasive ICP monitoring, and those with non-aneurysmal SAH were excluded. Informed consent was obtained from all eligible patients or their legal representatives if the patient was unable to provide consent. The study was performed in accordance with the ethical committee of our institution and the Declaration of Helsinki. The study was approved by the local institutional review board of the Ethics Committee (approval number: 16/9/20, date: September 2020, study title: “Impact of optimal cerebral perfusion pressure (CPPopt)—targeted hemodynamic therapy for delayed cerebral ischemia on delayed infarctions after aneurysmal subarachnoid hemorrhage”).

Ruptured intracranial aneurysms were detected using computed tomography angiography (CTA) and/or digital subtraction angiography (DSA). Aneurysm treatment was conducted within 48 h after ictus by microsurgical clipping or endovascular coiling after interdisciplinary consensus. All patients diagnosed with aSAH were admitted to the intensive care unit (ICU) for at least 14 days, and all treatments were aligned with current treatment guidelines^12^. Owing to their poor clinical condition, all patients in this study were sedated and mechanically ventilated. Because neurological examination is limited in patients with severe aSAH, a predefined imaging-based protocol including CT perfusion (CTP) and CTA on days 3 and 7 after ictus was routinely applied in comatose and sedated patients^22^. Intravenous nimodipine was administered continuously for cerebral vasospasm prophylaxis, and the blood flow velocity of the middle cerebral artery was measured daily using transcranial Doppler sonography (TCD). An increase in blood flow velocity > 120 cm/s (mean) and a Lindegaard index > 3 were considered TCD-vasospasm. If a patient was diagnosed with TCD-vasospasm, additional imaging studies using CTA and CTP were performed to verify the diagnosis. If the CTA demonstrated angiographic vasospasm (narrowing of at least 50% (moderate vasospasm) or > 75% (severe vasospasm) compared to the vessel diameter on admission) with an accompanying perfusion deficit on CTP, IHT was initiated by receiving intravenous norepinephrine with a target systolic blood pressure of 160–180 mmHg. The start date and duration of IHT were documented.

### Monitoring and data acquisition

An arterial line, usually through the radial artery, was placed for continuous monitoring of ABP. Mean arterial pressure was monitored using a standard pressure monitoring kit (DTXPLUS^®^ Becton Dickinson Infusion Therapy Systems Inc.). For multimodal data acquisition, IntelliSpace Critical Care and Anesthesia (ICCA) ICU software was used. For ICP monitoring, an intraparenchymal ICP probe (Codman Microsensors ICP Transducer; Codman & Shurtleff, Inc.) was inserted into the right frontal lobe. The ABP and ICP were continuously monitored using a Philips IntelliVue MX monitor. The signals were sampled at a frequency of 60 Hz and processed on a monitor with an integrated algorithm that suppressed the influence of pulse and respiratory frequency wave components. The averaged 10-second samples were transmitted to the server of the documentation system. Data were extracted and used for analysis in this study. PRx was calculated as correlation coefficients within a 4-hour time window, between 16 samples of time-averaged (15 min) data points of ICP and MAP. Hence, the calculated PRx in this study corresponds to long-PRx^23^. The PRx values range from − 1 to + 1, where negative values or zero represent an intact CA and positive values represent an impaired CA. By plotting PRx versus CPP, the CPP correlating to the lowest value of PRx was identified as CPPopt. Regression analysis (second-order quadratic regression) was conducted as a curve-fitting method, where the convex point of the curve corresponding to the lowest PRx value was determined as CPPopt, which was found in most cases (76.9%). In the case of a descending or ascending curve, the CPP corresponding to the lowest PRx value was determined as CPPopt.

### Primary outcome parameters

Delayed cerebral infarction was defined according to the consensus definition by Vergouwen et al.^24^. A CT scan was conducted within 24 h after aneurysm occlusion to exclude treatment-associated cerebral infarction. All other newly diagnosed cerebral infarctions were considered delayed cerebral infarctions. Functional outcomes were assessed according to the modified Rankin Scale (mRS) at the 3-month follow-up; an mRS score of ≤ 3 was considered a favorable outcome.

### Statistical analysis

IBM SPSS Statistics for Windows (Version 26.0, Armonk, NY, IBM Corp.) and GraphPad Prism software (Version 9, GraphPad Software, San Diego, CA, USA) were used for statistical analysis. Categorical data were presented as frequency and percentages, and continuous parameters as mean ± standard deviation (SD) or median and range. The Shapiro-Wilk test was used to assess the normality of the data distribution. The deviation of the actual CPP (i.e., the measured CPP) from CPPopt and the time spent with CPP < CPPopt were calculated. As the data were not normally distributed, nonparametric tests were conducted. The Mann-Whitney U test was used to assess differences between groups (IHT vs. no IHT, delayed cerebral infarction vs. no delayed cerebral infarction, and favorable vs. unfavorable outcomes). Additionally, two groups, patients with CPP near CPPopt (deviation < 5 mmHg) and patients with CPP < CPPopt, were defined and compared regarding outcome parameters. The days with measured CPP lower than the calculated CPPopt were summed up and defined as the time spent with lower CPP compared to the CPPopt. Regression analysis was performed to assess an association of the deviation of CPP from the calculated CPPopt with delayed cerebral infarctions. For the comparison of categorical variables, the Chi-square and Fisher’s exact test were used as appropriate. Statistical significance was defined as a *p*-value < 0.05.

## Results

### Patient characteristics

**Fig. 1:**
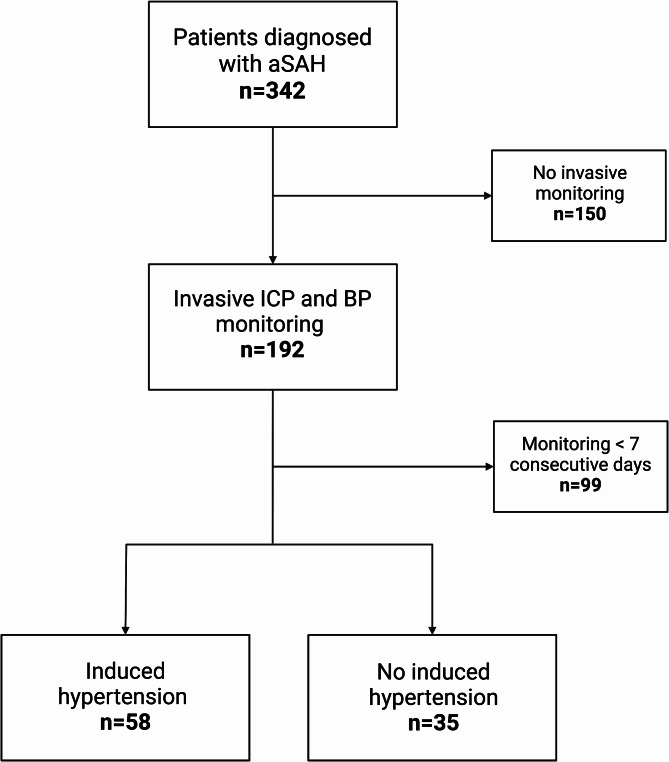
Study flowchart.

A total of 324 consecutive patients with aSAH were potentially eligible for this study. 28% (93/324) of these patients underwent invasive ICP and ABP monitoring and were included in further analysis (Fig. 1). The mean age was 54.2 ± 11.8 years. A higher WFNS grade (4–5) was observed in 64% (37/93) of patients. The clinical status of the included patients with an initially low WFNS grade worsened within the first few hours, with the consequence of intubation and the necessity of invasive ICP monitoring. All included patients were diagnosed with Fisher grade ≥ 3. The baseline characteristics of the study population are summarized in Table 1 and physiological parameters in Table 2.

**Table 1:** Baseline characteristics.

	*n* = 93	No IHT, *n* (%) *n* = 35 (38%)	IHT, *n* (%) *n* = 58 (62%)	*p*-value
Age				
Mean ± SD	54.2 ± 11.8	56.1 ± 12.8	54.5 ± 11.0	
(range)	(28–80)	(28–80)	(29–76)	*p* = 0.35
Sex				
M : F	60:33	22:13	38:20	*p* = 0.80
Hunt & Hess				
≤ 3	35 (38%)	9 (26%)	26 (45%)	
> 3	58 (62%)	26 (74%)	32 (55%)	*p* = 0.07
WFNS				
≤ 3	33 (35%)	12 (34%)	21 (36%)	
> 3	60 (65%)	23 (66%)	37 (64%)	*p* = 0.85
Fisher				
III	33 (35%)	7 (20%)	26 (45%)	
IV	60 (65%)	28 (80%)	32 (55%)	*p* = 0.02
Treatment				
Clipping	52 (56%)	18 (51%)	34 (59%)	
Coiling	41 (44%)	17 (49%)	24 (41%)	*p* = 0.50
DCI				
No	67 (72%)	27 (77%)	40 (69%)	
Yes	26 (28%)	8 (23%)	18 (31%)	*p*=0.40
mRS at 3 months				
Favorable	55 (59%)	18 (51%)	37 (64%)	*p*=0.41
Unfavorable	32 (34%)	15 (43%)	17 (29%)	
n.a.	6 (7%)	2 (6%)	4 (7%)	

IHT, induced hypertension; DCI, delayed cerebral infarction; WFNS, World Federation of Neurosurgical Societies; mRS, modified Rankin scale.

**Table 2:** Physiological parameters.

	MAP (mmHg)	Systolic BP (mmHg)	CPP (mmHg)	ICP (mmHg)	TCD BFV right (cm/s)	TCD BFV left (cm/s)
Day 1	86.2 (67.2–111.1)	122.6 (83.5–181.0)	76.8 (57.2–97.7)	7 (1–26)	51 (20–152)	54 (23–145)
Day 2	83.6 (70.3–106.0)	124.3 (97.5–158.3)	75.6 (63.9–105.0)	6.5 (0–26)	67 (24–140)	64 (25–138)
Day 3	84.3 (74.8–115.4)	128.4 (102.6–162.4)	77.5 (53.9–122.6)	8 (0–21)	76 (46–150)	74 (34–166)
Day 4	84.8 (71.6–120.5)	133.3 (105.3–183.4)	82.4 (60.6–132.9)	7 (1–21)	90 (32–210)	94 (34–220)
Day 5	90.7 (66.3–129.1)	139.2 (109.2–185.6)	84.2 (60.9–150.8)	7 (1–30)	96 (32–210)	96 (26–200)
Day 6	92.6 (70.0–135.4)	144.3 (115.9–182.8)	88.6 (66.3–129.3)	7.5 (1–26)	96 (38–288)	88 (36–220)
Day 7	97.0 (71.8–139.1)	148.9 (108.9–185.5)	90.2 (66.2–128.4)	8 (1–21)	101 (40–178)	88 (45–236)
Day 8	99.5 (73.6–132.8)	150.5 (114.9–189.1)	90.0 (61.6–128.9)	7 (2–22)	94 (23–185)	99 (34–186)
Day 9	99.6 (72.4–140.9)	151.7 (120.4–204.8)	92.4 (57.7–135.1)	7 (1–26)	80 (17–180)	88 (26–194)
Day 10	99.4 (72.0–136.9)	155.1 (117.4–197.3)	91.1 (21.7–122.2)	6 (1–57)	81 (20–200)	86 (20–194)
Day 11	99.3 (71.6–131.4)	150.5 (116.1–198.5)	90.0 (43.4–126.0)	7 (1-67)	88 (26–200)	86 (28–166)
Day 12	97.8 (73.0–139.4)	147.7 (119.1–193.0)	89.8 (59.4–127.0)	7 (1–52)	90 (38–168)	89 (38–190)
Day 13	98.5 (74.0–130.4)	148.5 (106.9–184.6)	90.7 (55.8–127.6)	7 (1–22)	95 (37–180)	86 (24–180)
Day 14	97.8 (69.9–131.5)	146.3 (111.0–192.7)	87.2 (54.2–119.7)	7 (1 -39)	84 (40–210)	84 (13–190)

Physiological measurements on each of the 14 monitored study days. Data are presented as median (range).

BFV, blood flow velocity; BP, blood pressure; ICP, intracranial pressure; MAP, mean arterial pressure; TCD, transcranial doppler sonography.

### Induced hypertension

**Fig. 2:**
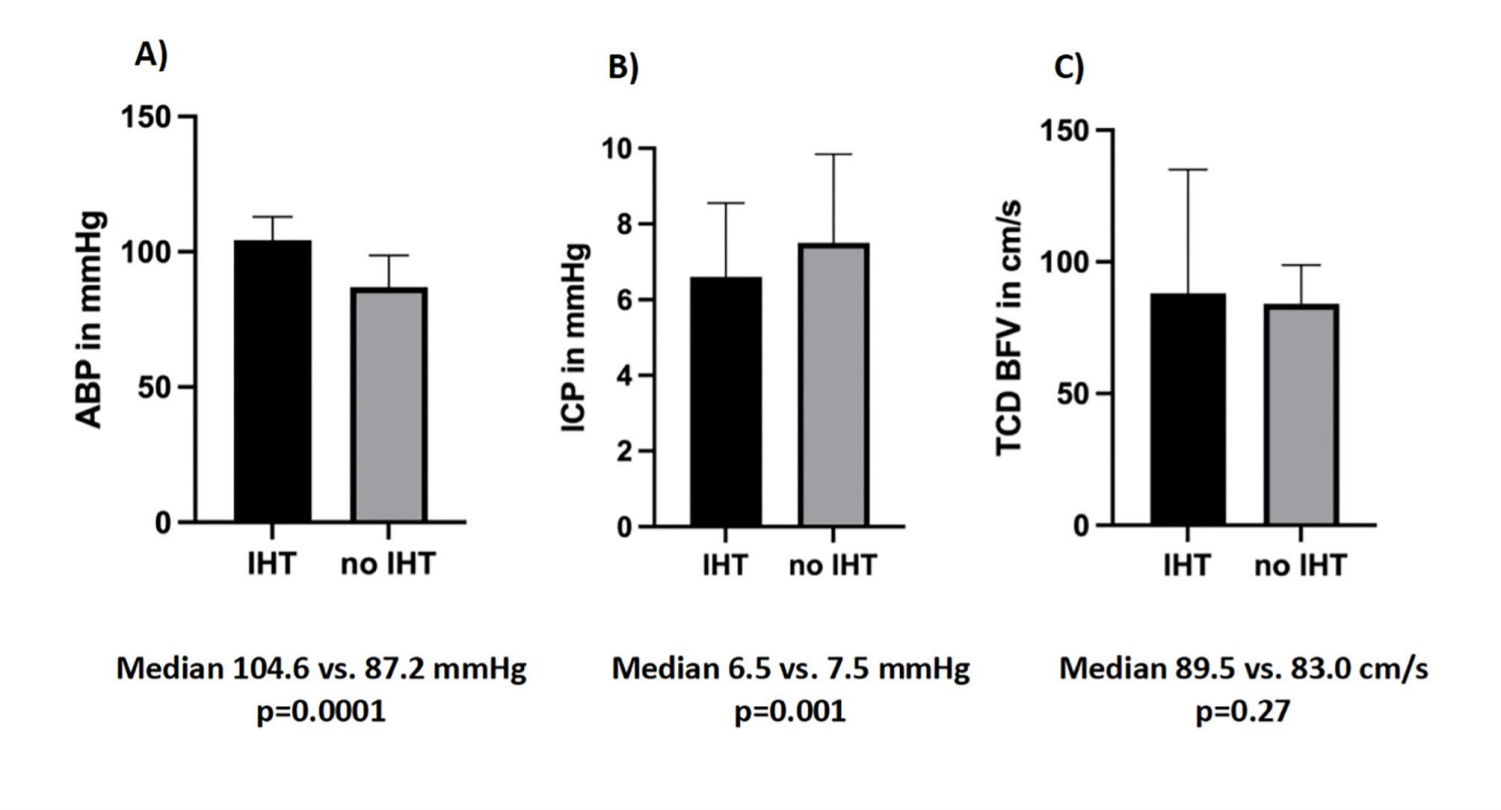
Comparison of average values of arterial blood pressure (ABP) showed significantly higher values in the IHT group than in the no IHT group **(graph A)**, average values of intracranial pressure (ICP) were significantly higher in the no IHT group than in the IHT group **(graph B)**, and average values of blood flow velocities (BFV) measured by transcranial Doppler sonography (TCD) showed higher values in the IHT group, but the difference was not statistically significant **(graph C)**.

IHT was performed in 62% (58/93) of patients, with an average start on day 6 and a median duration of 5 days (95%CI 3–7). The median CPP in the patient group without IHT (79.3 mmHg, 95% 76–82.5 mmHg) was significantly lower than the CPP in the patient group with IHT (99.5 mmHg, 95%CI 97.2–106.1 mmHg), *p* < 0.0001. The median CPPopt in the patient group without IHT (79.3 mmHg, 95%CI 76.8–83 mmHg) was also significantly lower than the median CPPopt in the patient group with IHT (103 mmHg, 95%CI 98.2–107.7 mmHg), *p* < 0.0001. Figure 2 additionally shows a comparison of average ABP, ICP, and TCD values for the first 14 days after ictus between both groups (IHT vs. no IHT).

### Delayed infarctions and functional outcome

**Fig. 3:**
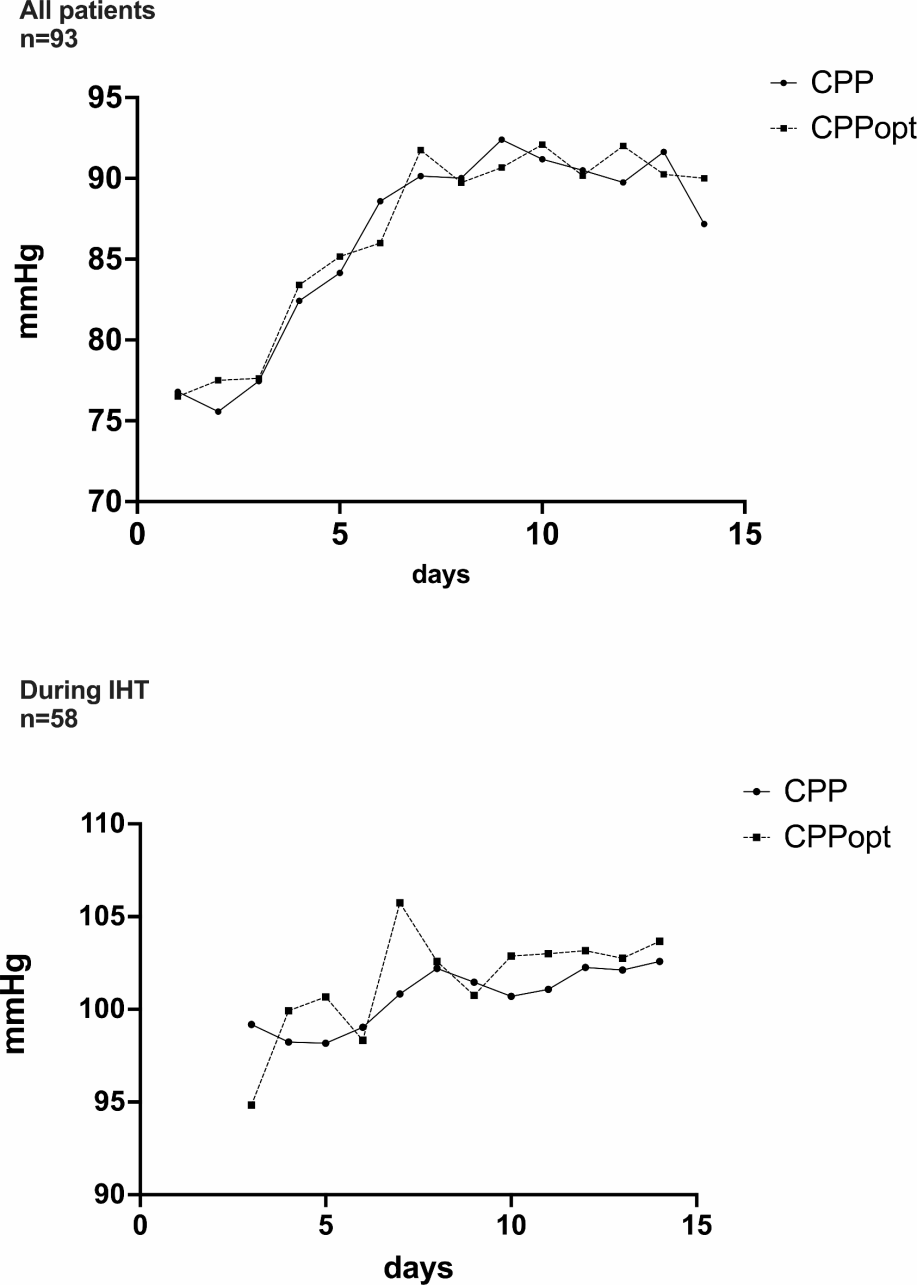
Time trend of cerebral perfusion pressure (CPP) values and optimal cerebral perfusion pressure (CPPopt) values within the first 14 days after ictus in the entire patient cohort (upper graph) and in the group treated with induced hypertension (IHT) during the time mean arterial pressure was increased by the administration of norepinephrine (lower graph). The lines represent the mean values (for each day).

Delayed infarction was detected in 31% (18/58) of patients treated with IHT and in 23% (8/35) of patients without IHT. Patients with delayed cerebral infarctions spent an average of 57% and patients without delayed cerebral infarctions spent an average of 33% of the time during which IHT was performed, with CPP values lower than the calculated CPPopt. A longer time spent with a measured CPP lower than the CPPopt during IHT was significantly associated with delayed cerebral infarctions (Y-intercept = 2.692, 95%CI 2.148 to 3.237, *p* = 0.002). Additionally, a trend towards a higher deviation of CPP from CPPopt during IHT in patients with delayed cerebral infarctions compared to those without delayed cerebral infarctions (18.6 mm Hg vs. 7.65 mmHg, *p* = 0.09) was demonstrated. In the patient group without IHT, delayed cerebral infarctions was also associated with higher deviations of measured CPP from calculated CPPopt (4.1 mmHg vs. 0.76 mmHg, *p* = 0.001). The time trends of CPP and CPPopt in the entire patient cohort and in the IHT group are shown in Fig. 3.

**Fig. 4:**
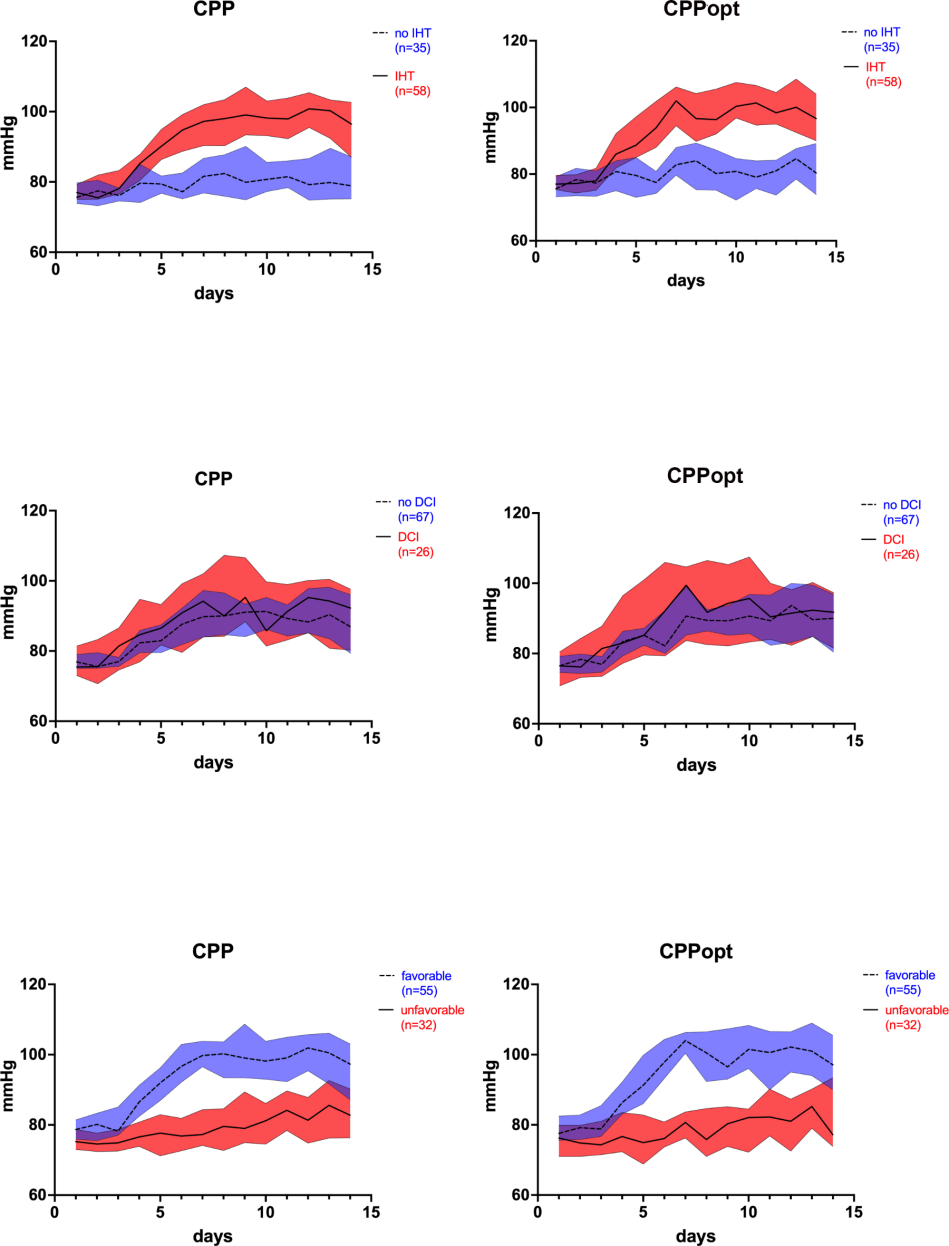
CPP and CPPopt values over the course of 14 days in patients with IHT and without IHT, patients with DCI and without DCI, and in patients with favorable and unfavorable outcome. The lines represent the mean values (for each day), and the colored areas represent the 95% confidence interval.

The median mRS score at the 3-month follow-up in the entire patient cohort was 1 (range: 0–6). In the patient group with IHT, higher deviations of the measured CPP from the calculated CPPopt were found in patients with unfavorable outcomes (average deviation 2.4 mmHg vs. 0.32 mmHg, *p* = 0.04). A higher deviation of the measured CPP from the CPPopt during IHT was also associated with a higher mRS score (worse outcome) (*r* = 0.32, *p* = 0.02). In patients without IHT higher deviations of measured CPP from calculated CPPopt also led to a worse outcome (average deviation 2.7 mmHg vs. 0.32 mmHg, *p* = 0.0007). In Fig. 4, the time course of CPP and CPPopt values over a time course of 14 days after ictus for patients with and without delayed cerebral infarctions, and patients with favorable and unfavorable outcomes are displayed.

## Discussion

In this study, the deviation of CPP from CPPopt during IHT was calculated in patients with aSAH, and its impact on the incidence of delayed infarctions and functional outcomes was evaluated. Deviations of measured CPP from the calculated CPPopt during IHT were significantly correlated with the development of delayed infarctions and poor outcomes in patients with aSAH. Currently, IHT is performed as a last resort intervention for the treatment of delayed cerebral ischemia in patients with aSAH, although its effect is primarily based on observational studies^12^. The HIMALAIA (Hypertension Induction in the Management of AneurysmaL subarachnoid Hemorrhage with secondary IschemiA) trial was the first RCT to investigate the effect of IHT on delayed cerebral ischemia, which was prematurely terminated due to slow recruitment^25^. The data analysis of the 41 included patients demonstrated a lack of effect on cerebral perfusion and did not provide any evidence to support IHT^25^. Nevertheless, recent literature suggests that IHT increases CBF in areas with the lowest perfusion, which is of clinical interest, as these areas can progress to infarction. Furthermore, an increase in MAP may improve CBF during disrupted autoregulation^26^. However, the extent to which MAP should be elevated by vasopressor administration to improve cerebral perfusion in individual patients remains unclear. Current guidelines do not recommend a specific CPP cutoff to prevent hypoperfusion in the acute phase of aSAH^12^. At our institution, a systolic blood pressure of 160–180 mm Hg is usually targeted when induced hypertension is indicated. The median CPP measured during IHT in our study cohort was 99 mmHg (range, 73–129 mmHg). However, this treatment approach does not consider the functional status of CA in individual patients, which can be disturbed to varying extents, depending on the severity of aSAH. Previous studies have demonstrated that impaired CA represents a key component of secondary brain injury after aSAH and is also an independent predictor of ischemic events and poor outcomes^21,27,28^. Owing to the increasing acknowledgement of CA as an important contributor to the pathophysiology of brain injury, the concept of optimal CPP (CPPopt)-targeted treatment has emerged in recent years and is currently under investigation in the setting of different cerebral pathologies. While most of the evidence has been obtained for patients with TBI, only a few studies have evaluated this concept in the context of aSAH. Several retrospective studies have demonstrated that a smaller deviation of CPP from CPPopt was associated with better clinical outcome in TBI^15–17^. This concept is currently under investigation in a randomized controlled trial (COGiTATE) in a patient population with TBI^29^. Although CPPopt targets may be beneficial in patients with TBI, where a CPP between 60 and 70 mmHg was previously demonstrated to be associated with favorable outcomes, whereas a CPP > 70 mmHg led to worse clinical outcomes^30^, the opposite may apply in the setting of aSAH. Additionally, studies have demonstrated that CPPopt changes over time and differs individually among aSAH patients, concluding that a universal “one-size-fits-all” CPPopt threshold may not be the appropriate treatment approach in aSAH patients^19,21,28^. The current literature indicates that lower CPP values correlate with a higher incidence of ischemic events and unfavorable outcomes in aSAH. Jaeger et al. found that an increase in the time spent with a CPP below the calculated CPPopt was associated with a worse outcome^21^. Svedung et al. also reported that CPPopt was higher in patients with favorable outcomes^28^. In our cohort, a larger deviation in absolute CPP from the PRx-derived CPPopt during IHT was associated with worse clinical outcomes. However, Svedung et al. could not demonstrate a correlation between deviations of absolute CPP from the CPPopt with poor outcome in their study. In our cohort, a larger deviation in the measured CPP from CPPopt and lower CPPopt during IHT were associated with a higher incidence of delayed infarctions. Bijlenga et al. also demonstrated that optimal CPP was higher during cerebral vasospasm^19^. Based on these results, Schmidt et al.^31^ found that a CPP level < 70 mm Hg (even when CPP was between 60 and 70 mmHg) was associated with a higher risk of brain tissue hypoxia and metabolic crisis in poor grade aSAH-patients. Overall, this suggests that a more aggressive approach, with higher CPP values, may be beneficial for aSAH. However, further prospective trials are necessary to confirm these findings. Although there are certain risks associated with continuous hyperdynamic therapy, such as rebleeding, cerebral edema, increased ICP, posterior reversible encephalopathy syndrome, and reversible leukoencephalopathy, there are no guidelines to help clinicians define effective and safe hypertension limits. Besides cerebral injury, the administration of systemic catecholamines can lead to myocardial ischemia and acute renal and pulmonary complications^32^. Therefore, the goal of further studies will be to identify patients who will most likely benefit from hyperdynamic therapy. Invasive monitoring of PRx and calculation of CPPopt may be useful clinical tools to guide the timing and extent of IHT.

### Limitations of the study

Due to the retrospective study design and the limited number of patients included, the findings presented here do not allow general conclusions regarding causation. Due to a selection of the patient population with the presence of invasive ICP monitoring as one of the main inclusion criteria, all included patients had an EVD according to the management protocol in our hospital for comatose/sedated aSAH patients. The presence of EVD may have an influence on the PRx calculation, hence, it cannot be excluded that PRx calculation in a patient population without EVD could lead to different results, which is a limitation of the study. Although a predefined protocol was followed for the treatment of patients with aSAH, IHT was indicated on an individual basis. The calculations of PRx and CPPopt were based on data recorded from the monitoring and documentation system in the intensive care unit. PRx was calculated within a 4-hour window, hence, the calculated PRx in our study corresponds to the long-PRx, as described by Lang et al. 2015 ^23^. Additionally, overestimation or underestimation of the calculated CPPopt cannot be excluded in the case of a descending/ascending curve, instead of a U-shaped curve. Hence, it needs to be acknowledged that these methodological differences might have influenced the findings of our study, which is a limitation and must be considered during the interpretation of the results. However, a clinically oriented approach was followed, using data acquired via a widely available monitoring and documentation system in intensive care units.

## Conclusions

Longer time spent with measured CPP < CPPopt during IHT increased the risk of developing delayed cerebral infarctions, and a larger deviation of the measured CPP from the optimal CPP during IHT was associated with worse outcomes. According to our results, the estimation of CPPopt seems to be useful for the guidance of IHT and preservation of optimal cerebral perfusion during acute management of patients with severe aSAH.

## Data Availability

All available data without compromising individual privacy are presented in the manuscript.
